# 

**Published:** 1988-05

**Authors:** 


JOURNAL

### BRISTOL MEDICO-CHIRURGICAL JOURNAL

VOLUME 103(ii) MAY 1988

### Magnetic Resonance Imaging

NATIONAL
LIBRARY
AUG 22 1988
OF
MEDICINE

THE MEDICAL JOURNAL OF THE SOUTH WEST

